# Evolution of cytokinesis-related protein localization during the emergence of multicellularity in volvocine green algae

**DOI:** 10.1186/s12862-017-1091-z

**Published:** 2017-12-06

**Authors:** Yoko Arakaki, Takayuki Fujiwara, Hiroko Kawai-Toyooka, Kaoru Kawafune, Jonathan Featherston, Pierre M. Durand, Shin-ya Miyagishima, Hisayoshi Nozaki

**Affiliations:** 1 Department of Biological Sciences, Graduate School of Science, University of Tokyo, 7-3-1 Hongo, Bunkyo-ku, Tokyo, 113-0033 Japan; 2 Department of Cell Genetics, National Institute of Genetics, 1111 Yata, Mishima, Shizuoka 411-8540 Japan; 3 Department of Life Science and Technology, School of Life Science and Technology, Tokyo Institute of Technology, 2-12-1 Ookayama, Meguro-ku, Tokyo, 152-8550 Japan; 4 Evolutionary Studies Institute, University of the Witwatersrand, Johannesburg, 2000 South Africa; 5 Agricultural Research Council, Biotechnology Platform, Pretoria, 0040 South Africa; 6 Department of Ecology and Evolutionary Biology, University of Arizona, Tucson, AZ 85721 USA

**Keywords:** Multicellularity, Volvocine algae, *Tetrabaena socialis*, DRP1

## Abstract

**Background:**

The volvocine lineage, containing unicellular *Chlamydomonas reinhardtii* and differentiated multicellular *Volvox carteri*, is a powerful model for comparative studies aiming at understanding emergence of multicellularity. *Tetrabaena socialis* is the simplest multicellular volvocine alga and belongs to the family Tetrabaenaceae that is sister to more complex multicellular volvocine families, Goniaceae and Volvocaceae. Thus, *T. socialis* is a key species to elucidate the initial steps in the evolution of multicellularity. In the asexual life cycle of *C. reinhardtii* and multicellular volvocine species, reproductive cells form daughter cells/colonies by multiple fission. In embryogenesis of the multicellular species, daughter protoplasts are connected to one another by cytoplasmic bridges formed by incomplete cytokinesis during multiple fission. These bridges are important for arranging the daughter protoplasts in appropriate positions such that species-specific integrated multicellular individuals are shaped. Detailed comparative studies of cytokinesis between unicellular and simple multicellular volvocine species will help to elucidate the emergence of multicellularity from the unicellular ancestor. However, the cytokinesis-related genes between closely related unicellular and multicellular species have not been subjected to a comparative analysis.

**Results:**

Here we focused on dynamin-related protein 1 (DRP1), which is known for its role in cytokinesis in land plants. Immunofluorescence microscopy using an antibody against *T. socialis* DRP1 revealed that volvocine DRP1 was localized to division planes during cytokinesis in unicellular *C. reinhardtii* and two simple multicellular volvocine species *T. socialis* and *Gonium pectorale*. DRP1 signals were mainly observed in the newly formed division planes of unicellular *C. reinhardtii* during multiple fission*,* whereas in multicellular *T. socialis* and *G. pectorale*, DRP1 signals were observed in all division planes during embryogenesis.

**Conclusions:**

These results indicate that the molecular mechanisms of cytokinesis may be different in unicellular and multicellular volvocine algae. The localization of DRP1 during multiple fission might have been modified in the common ancestor of multicellular volvocine algae. This modification may have been essential for the re-orientation of cells and shaping colonies during the emergence of multicellularity in this lineage.

**Electronic supplementary material:**

The online version of this article (10.1186/s12862-017-1091-z) contains supplementary material, which is available to authorized users.

## Background

**Fig. 1:**
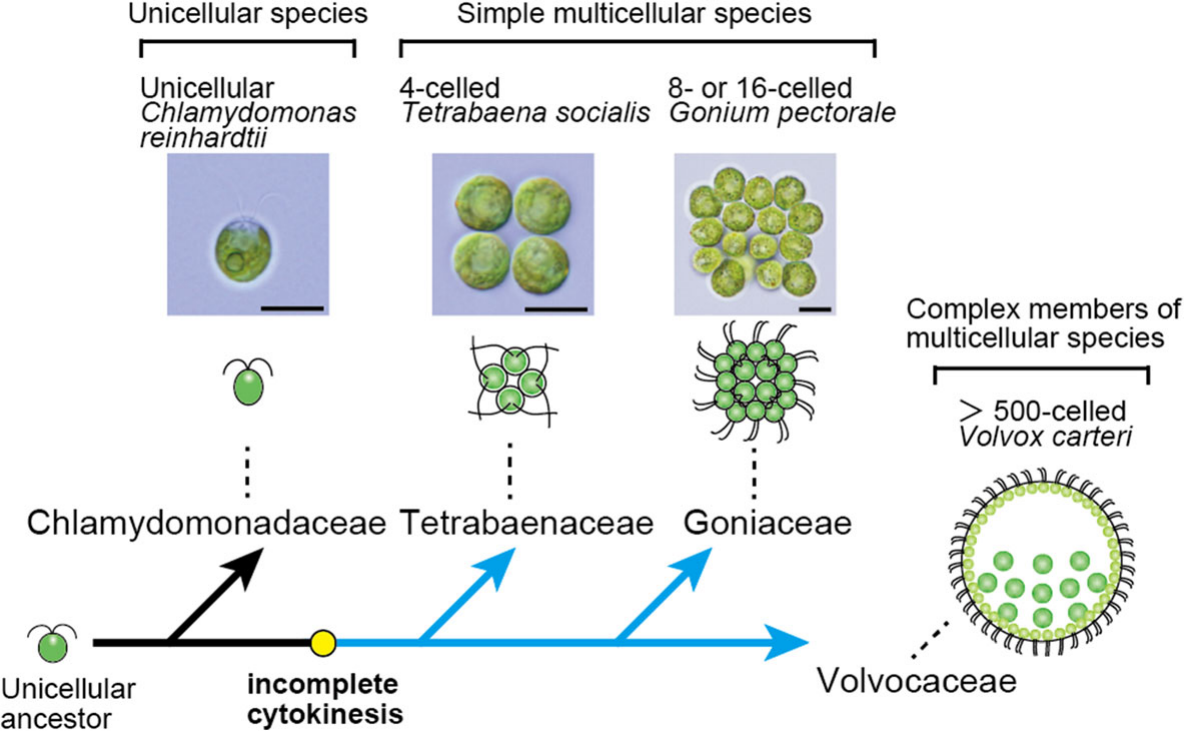
Simplified representation of volvocine phylogeny and evolution of incomplete cytokinesis, focusing the three volvocine species examined in the present study. The phylogeny is based on previous studies [3, 6, 7]. Scale bars: 10 μm

The transition to multicellularity is one of the most compelling events during the evolution of life and has occurred more than 25 times in distinct eukaryotic lineage [1]. To fully understand these transitions comparative biological studies are essential. Major complex multicellular groups such as metazoans and land plants emerged from unicellular ancestors that existed approximately 600 to 1000 million years ago [2]. There are few extant species that represent the initial features of multicellular ancestors or transitional forms from unicellular to multicellular within the lineages closely related to metazoans and land plants. In contrast, the multicellular volvocine green algae (Fig. 1) diverged from a unicellular ancestor only 200 million years ago [3]. In addition, there are many extant species representing various steps in the transition to multicellularity, which makes the volvocines unique as a model lineage.

The volvocine lineage (Fig. 1) includes unicellular *Chlamydomonas reinhardtii*, undifferentiated multicellular species like *Tetrabaena socialis* and *Gonium pectorale*, and differentiated multicellular species such as *Volvox carteri* [3–5]. The simplest multicellular species is four-celled *T. socialis* belonging to the Tetrabaenaceae, which is sister to the large clade composed of the remaining, more complex colonilal/multicellular volvocine algae (Goniaceae and Volvocaceae) (Fig. 1) [5–7]. *T. socialis* shares at least four common features with more complex multicellular volvocine members: incomplete cytokinesis, rotation of basal bodies, transformation of the cell wall to extracellular matrix, and modulation of cell number [3, 5]. Recently, whole nuclear genome analyses of *G. pectorale* [8] suggested that modifications of cell cycle regulation genes (duplication of *cyclin D1* gene and alterations in the retinoblastoma protein) occurred in the common ancestor of *G. pectorale* and *V. carteri* and that these modifications were the basis for genetic modulation of cell number. However, there is less information about more downstream molecules that actually participate in the formation of integrated multicellular individuals during embryogenesis.

During the asexual life cycles of unicellular and multicellular volvocine algae, reproductive cells perform successive divisions (rapid S/M phase alternating without G2 phase) known as multiple fission [9, 10]. The unicellular species *C. reinhardtii* forms 2^n^ (n: number of rounds of cell divisions) daughter cells depending on the size of the mother cell [11], whereas reproductive cells of multicellular volvocine members form 2^n^ daughter protoplasts that are regulated by mother cell size and genetic control [4]. These daughter protoplasts are connected to one another by cytoplasmic bridges, which are important for the arrangement of cells within the daughter colony of multicellular volvocine algae [4] like *T. socialis* [5], *G. pectorale* [12] and *V. carteri* [13]. Considering that both of these multicellular member-specific traits (modulation of daughter cell number and incomplete cytokinesis) are recognized in the tetrabaenacean species *T. socialis*, comparative molecular analyses of multiple fission between unicellular and multicellular forms is essential to understand the initial steps to multicellularity in this lineage.

Various cytokinesis-related genes are characterized in metazoans [14] and land plants [15]. Modes of cytokinesis are variable: mother cells divide into two daughter cells by fission in metazoans, whereas two daughter cells are produced by a cell plate newly formed in land plant mother cells [16]. However, there are some common molecules associated with cytokinesis in metazoans and land plants [14, 15] such as dynamin-related protein (DRP). Dynamin was originally described as a microtubule-binding protein that was isolated from bovine brain extracts [17]. Dynamin homologs are often categorized as classical or conventional dynamins, which have five distinct domains: GTPase domain, middle domain, pleckstrin-homology domain, GTPase effecter domain (GED), and proline-rich domain [18, 19]. The dynamin superfamily contains additional members that lack a pleckstrin-homology domain and/or proline-rich domain, or have additional domains that are not present in classical dynamins. These members are defined as dynamin-related proteins (DRPs) [19]. In metazoans and land plants, several dynamins and DRPs play important roles in cytokinesis [18, 19]. For example, in *Arabidopsis thaliana*, DRP1A and DRP1E are localized in the cell plate [20], DRP2B is co-localized with DRP1A in the cell plate during cytokinesis and functions in vesicle formation [21], and DRP5A is localized in the cell plate at the end of cell division [22]. The double mutant line *drp1a/drp1e* of *A. thaliana* is unable to accomplish the embryogenesis because of defects in cell wall formation [20].

In this study, we focused on the DRP1 homologs in the volvocine lineage to examine the contribution of cytokinesis-related genes to the initial stages of multicellularity. We determined the complete coding region of the *DRP1* homolog of the simplest multicellular species *T. socialis* (*TsDRP1*), and performed immunofluorescence microscopy with a newly raised anti-TsDRP1 antibody in unicellular *C. reinhardtii* and two colonial multicellular species *T. socialis* and *G. pectorale* (Fig. 1).

## Methods

### Strains and culture conditions

Three algal strains were used in this study: *C. reinhardtii* strain cw92 (CC-503, cell-wall deficient, distributed by the *Chlamydomonas* Resource Center) [23], *T. socialis* strain NIES-571 [5], and *G. pectorale* strain 2014–0520-F1–4 (a sibling strain of *plus* and *minus* strains used previously [24]). *C. reinhardtii* strain cw92 was cultured synchronously in 300 mL tris-acetate-phosphate medium [25] in a silicon-capped 500 mL flask with aeration at 25 °C, on a light: dark cycle 12 h: 12 h under cool-white fluorescent lamps at an intensity of 110–150 μmol∙m^−2^∙s^−1^. *T. socialis* strain NIES-571 was cultured synchronously in 300 mL standard *Volvox* medium [26] in a silicon-capped 500 mL flask with aeration at 20 °C, on a light: dark cycle 12 h: 12 h under cool-white fluorescent lamps at an intensity of 110–150 μmol∙m^−2^∙s^−1^ [5]. *G. pectorale* strain 2014–0520-F-4 was cultured synchronously in 300 mL standard *Volvox* medium [26] in a silicon-capped 500 mL flask with aeration at 20 °C, on a light: dark cycle 12 h: 12 h under cool-white fluorescent lamps at an intensity of 130–180 μmol∙m^−2^∙s^−1^. To evaluate the synchrony, percentages of dividing cells were monitored 15–16 times during 24 h.

### Identification of *TsDRP1* gene

To determine the complete coding region of *TsDRP1*, partial coding sequences were obtained from our ongoing *T. socialis* strain NIES-571 genome assembly by a TBLASTN search using *C. reinhardtii* DRP1 (CrDRP1) as a query, and *TsDRP1* specific primers (Additional file 1: Table S1) were designed based on the sequences. Polyadenylated mRNA of *T. socialis* was isolated with Dynabeads oligo (dT)_25_ (Thermo Fisher Scientific, Waltham, MA, USA) and reverse transcribed with Superscript III reverse transcriptase (Thermo Fisher Scientific) [24]. PCR was carried out using the synthesized cDNA as follows: 94 °C for 1 min, 35 cycles of 94 °C for 30 s, 60 °C for 30 s, and 72 °C for 2 min, followed by 72 °C for 5 min with *TaKaRa LA-Taq* with GC Buffer (Takara Bio Inc., Otsu, Japan) by using a thermal cycler GeneAmp PCR System 9700 (Thermo Fisher Scientific). Amplified DNA was purified with illustra GFX PCR DNA and Gel Band Purification Kits (GE Healthcare, Buckinghamshire, UK) and sequenced directly by an ABI PRISM 3100 Genetic Analyzer (Thermo Fisher Scientific) using a BigDye Terminator cycle sequencing ready reaction kit, v.3.1 (Thermo Fisher Scientific). The 5′ and 3′ ends of *TsDRP1* were determined by RACE using the GeneRacer™ kit (Thermo Fisher Scientific) according to the manufacturer’s protocol. Each synthesized RACE product was amplified with KOD FX Neo DNA polymerase (TOYOBO, Osaka, Japan). PCR was carried out as follows: 94 °C for 2 min, 5 cycles of 98 °C for 10 s and 74 °C for 1.5 min, 5 cycles of 98 °C for 10 s and 72 °C for 1.5 min, 5 cycles of 98 °C for 10 s and 70 °C for 1.5 min, 15 cycles of 98 °C for 10 s and 68 °C for 1.5 min, and followed by 68 °C for 7 min. Amplified DNA was purified and sequenced as described above. Domains of DRP1proteins were searched by using Pfam program (http://pfam.xfam.org) [27].

### Phylogenetic analyses

The amino acid sequences of DRP1 and DRP2 from six streptophytes (land plants and *Klebsormidium flaccidum*) and six chlorophytes (Additional file 1: Table S2) were collected from the genome databases in Phytozome (https://phytozome.jgi.doe.gov/pz/portal.html) and National Center for Biotechnology Information (https://www.ncbi.nlm.nih.gov/) by BLASTP and TBLASTN searches [28] using CrDRP1 and *A. thaliana* DRP2A as queries and aligned with the newly determined TsDRP1 by MAFFTv7 [29] server (http://mafft.cbrc.jp/alignment/server/). Phylogenetic analyses were performed using the maximum-likelihood and neighbor joining methods with PhyML [30] and MEGA 5.2.2 programs [31], respectively. The LG + I + G + F model was selected using ProtTest3 [32] optimized using Akaike information criteria. The bootstrap analyses were performed with 1000 replicates. Because DRP1 clade is sister to DRP2 clade [22], DRP2 sequences were treated as the outgroup in the present study.

### Preparation of antibodies

The antibody against *T. socialis* DRP1 (anti-TsDRP1 antibody) was raised in rabbits using the recombinant polypeptide. The cDNA sequence encoding the protein (corresponding to 11–597 positions of TsDRP1 amino acids; Additional file 2: Figure S1) was amplified by PCR using the primers listed in Additional file 1: Table S1. The PCR product was cloned into a pET100 expression vector (Thermo Fisher Scientific) and 6xHis fusion polypeptide was expressed in Rosetta (DE3) *Escherichia coli* cells, purified using a HisTrap HP column (GE healthcare). The purified polypeptide was subjected to SDS-PAGE, and gel slices containing the recombinant polypeptide were homogenized and injected into rabbits for antibody production (Kiwa Laboratory Animals. Co., Ltd., Wakayama, Japan). Antibodies were affinity-purified from the antisera by using the recombinant polypeptide coupled to a HiTrap NHS-activated HP column (GE Healthcare). Evaluation of the specificity of the anti-TsDRP1 antibody in *T. socialis*, *G. pectorale* and *C. reinhardtii* was performed by western blot analyses as described below (Additional file 2: Figure S2, Information S1).

### Western blot analyses

Expression of DRP1 proteins in the three volvocine species was analyzed by SDS-PAGE and western blot modified from a previous study [33]. Cells were harvested, suspended in SDS-sample buffer (100 mM dithiothreitol, 2% SDS, 10% glycerol, 0.005% Bromophenol blue in 62.5 mM Tris-HCl) and boiled for three minutes. The prepared samples were separated on an Any kD Mini-PROTEAN TGX precast gel (Bio-Rad, Hercules, CA, USA) and transferred onto a Hybond-P membrane (GE Healthcare, Uppsala, Sweden). The blotted membrane was blocked with 3% skim milk in TPBS [0.1% Tween 20 (Sigma Aldrich) in phosphate-buffered saline (PBS)] at 4 °C overnight. The blot was incubated with an anti-TsDRP1 antibody diluted 1: 2000 with 3% skim milk in TPBS for 1 h at room temperature and washed in 3% skim milk in TPBS. The membrane was incubated with a goat anti-rabbit IgG antibody conjugated to horseradish peroxidase (Jackson ImmunoResearch, WestGrove, PA, USA) diluted 1:2000 with 3% skim milk in TPBS for 1 h at room temperature and washed with TPBS. The protein signals were detected with Amersham ECL prime Western blotting detection reagent (GE Healthcare). Images were obtained by ChemiDoc XRS system (Bio-Rad) with Quantity One software (Bio-Rad).

To examine the expression of DRP1 at the protein level, five time-course samples were obtained from each synchronous culture of *C. reinhardtii*, *T. socialis*, and *G. pectorale*: a sample with the greatest number of dividing cells during 24 h (0 point), three hours before the 0 point (−3 point), six hours before the 0 point (−6 point), three hours after the 0 point (+3 point), and six hours after the 0 point (+6 point). The time-course samples were analyzed by SDS-PAGE and western blot as described above. For Coomassie brilliant blue staining as loading control, each duplicated SDS-PAGE gel was stained by EzStain AQua (ATTO, Tokyo, Japan).

### Indirect immunofluorescence microscopy

Fixation of *C. reinhardtii* was performed using a modified method in a previous study [34]. *C. reinhardtii* cells were attached to polyethyleneimine coated coverslips and fixed in −20 °C methanol for 5 min, transferred to fresh −20 °C methanol for 5 min and air-dried. The dried cells were incubated in PBS for 10 min. Subsequent blocking and antibody reactions were performed based on a previous study [5]. Immunostaining of *T. socialis* and *G. pectorale* were performed as described previously [5]. The anti-TsDRP1 antibody and a monoclonal anti-tubulin alpha antibody (clone YL1/2, Bio-Rad, Hercules, CA, USA), used as primary antibodies, were diluted 1: 500 with blocking buffer (0.11% Gelatin [Sigma Aldrich], 0.05% NaN_3_, 0.25% bovine serum albumin [Sigma Aldrich] in TPBS). Alexa Fluor 488 goat anti-rabbit IgG (H + L) (# A11008, Invitrogen, Carlsbad, CA, USA) and Alexa Fluor 568 goat anti-rat IgG (H + L) (# A11077, Invitrogen) were also diluted 1: 500 with the blocking buffer. Confocal and differential interference contrast (DIC) images were obtained with an FV-1200 (Olympus, Tokyo, Japan) and three serial images were merged by using Adobe Photoshop CS6 software (Adobe Systems Inc., San Jose, CA, US).

## Results

### Identification and characterization of *TsDRP1*

The full-length coding region of *TsDRP1* (1890 bp) was determined and the deduced amino acid sequence (629 amino acids) aligned with DRP1 homologs of *A. thaliana* (AtDRP1A), *C. reinhardtii* (CrDRP1), *G. pectorale* (GpDRP1), and *V. carteri* (VcDRP1) (Additional file 2: Figure S1). The DRP1 sequences were highly conserved within the volvocine algae: the identity of TsDRP1 with CrDRP1, GpDRP1, and VcDRP1 was 92%, 92%, and 91%, respectively. The GC contents of the volvocine *DRP1* coding region were higher (*CrDRP1*: 64.18%, *TsDRP1*: 65.12%, *GpDRP1*: 62.22%, and *VcDRP1*: 57.55%) than that of *AtDRP1A* (46.78%), which is consistent with their GC-rich genome compositions [8, 23, 35]. Three domains that characterize DRP1, GTPase domain, middle domain, and GED [19], were found in all DRP1 sequences of volvocine algae as well as AtDRP1A. The motifs for interactions to GTP (G1–4) [18] were conserved in AtDRP1A and volvocine DRP1 sequences (Additional file 2: Figure S1).

**Fig. 2:**
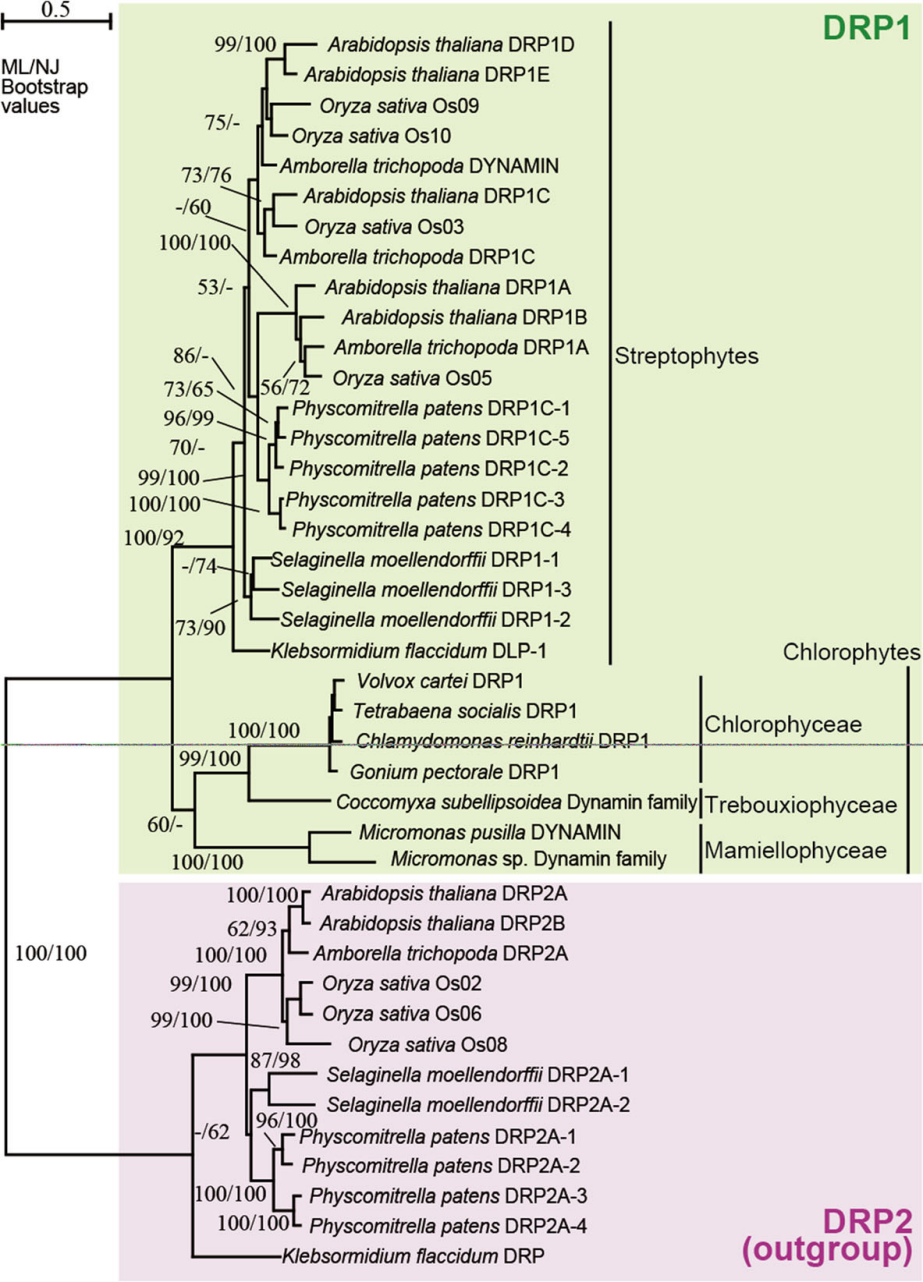
Maximum-likelihood (ML) tree of dynamin-related proteins. Bootstrap values (≥50%) for the ML and neighbor-joining (NJ) analyses are indicated left and right side, respectively. DRP2 sequences were used as outgroup. The scale bar corresponds to 0.5 amino acid substitutions per position

To confirm that DRP1 sequences of volvocine algae are orthologous to the DRP1 proteins of land plants, phylogenetic analyses were performed. All five land plants possessed several paralogs of *DRP1* genes, whereas each of the green algae (six chlorophytes and streptophyte *K. flaccidum*) possessed a single *DRP1* gene in the nuclear genome. The DRP1 clade was subdivided into two monophyletic groups corresponding to streptophytes and chlorophytes (Fig. 2). However, while the streptophyte clade was robustly resolved (with 92–100% bootstrap values), the monophyly of the chlorophytes was much less supported (with 60% bootstrap values in only maximum-likelihood method). The chlorophytes were composed of two robust clades, Chlorophyceae/Trebouxiophyceae and Mamiellophyceae with 99–100% bootstrap values. Within the former clade, four chlorophycean or volvocine DRP1 sequences formed a robust monophyletic group with 100% bootstrap values. These results were consistent with the phylogeny using multiple chloroplast genes [36–38], and indicate that *DRP1* genes of volvocine algae are orthologs of *DRP1* of streptophytes.

### Expression patterns of CrDRP1, TsDRP1 and GpDRP1 during the asexual life cycle

To examine the relationship between volvocine DRP1 and cytokinesis at a protein expression level, we performed western blot analyses using the anti-TsDRP1 antibody. The CrDRP1, TsDRP1, and GpDRP1 signals were detected as major bands at ~75 kDa with the anti-TsDRP1 antibody (Additional file 2: Figure S2). DRP1 signals were constitutively detected from all time-course samples of the three species (Additional file 2: Figure S3).

### Subcellular localization of DRP1 during the asexual life cycle

**Fig. 3:**
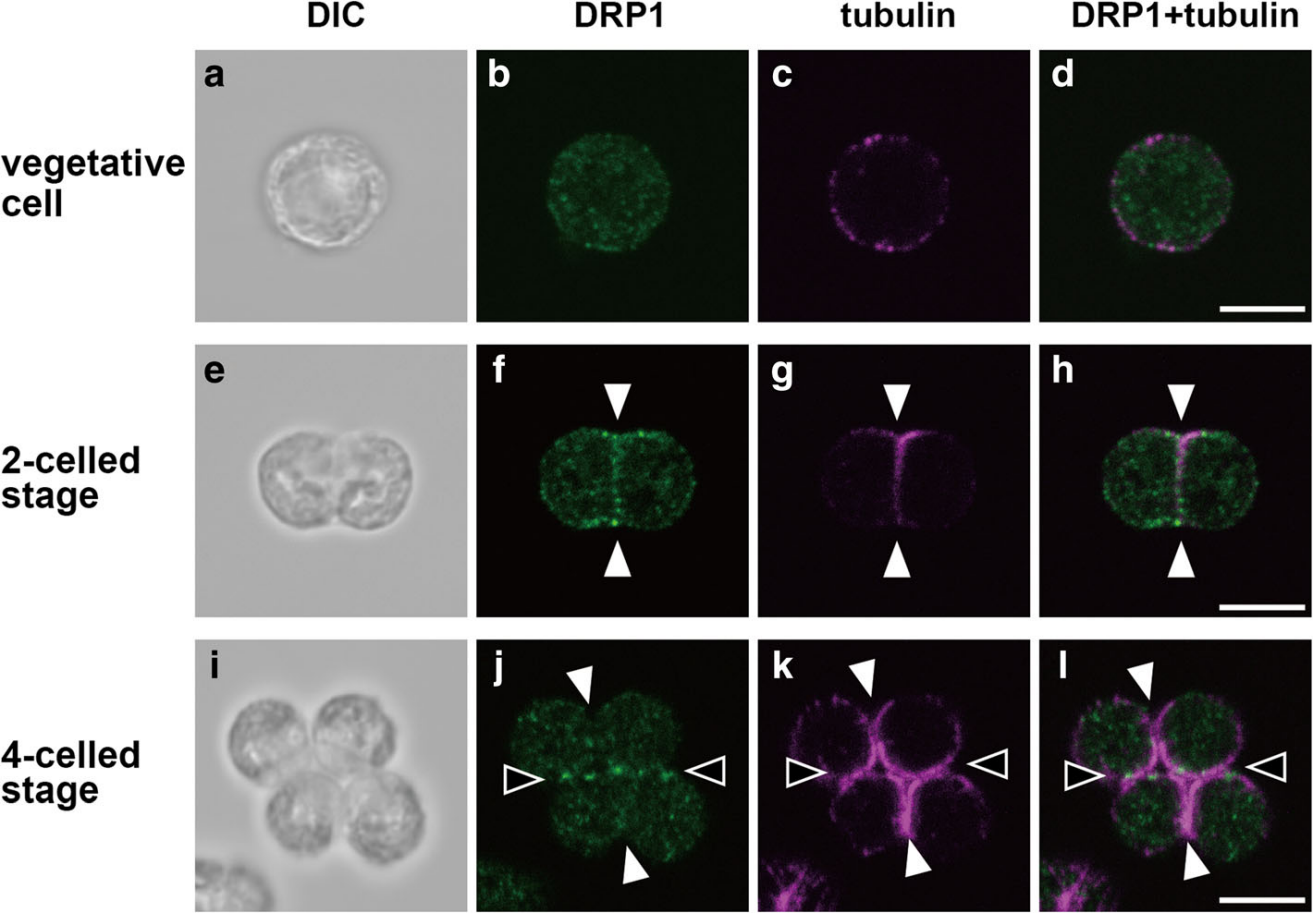
Immunofluorescence images of DRP1 localization in *Chlamydomonas reinhardtii*. Differential interference contrast (DIC) images (**a**, **e**, **i**), immunofluorescence images labeled with an anti-TsDRP1 (**b**, **f**, **j**) and an anti-tubulin alpha (**c**, **g**, **k**), and merged immunofluorescence images of DRP1 and tubulin (**d**, **h**, **l**) are shown. In vegetative cells, DRP1 was localized to cytoplasm (**b**-**d**). DRP1 was localized to a first division plane of two-celled embryo (white arrowheads in **f**-**h**) and second division planes of four-celled embryo (black arrowheads in **j**-**l**) mainly. Note that DRP1 signals localized to the first division plane of the two-celled embryo (**f**) become weak in the four-celled stage (**j**). The first and second division planes in the four-celled stage were estimated by relative positions of pyrenoids. Scale bars: 5 μm

**Fig. 4:**
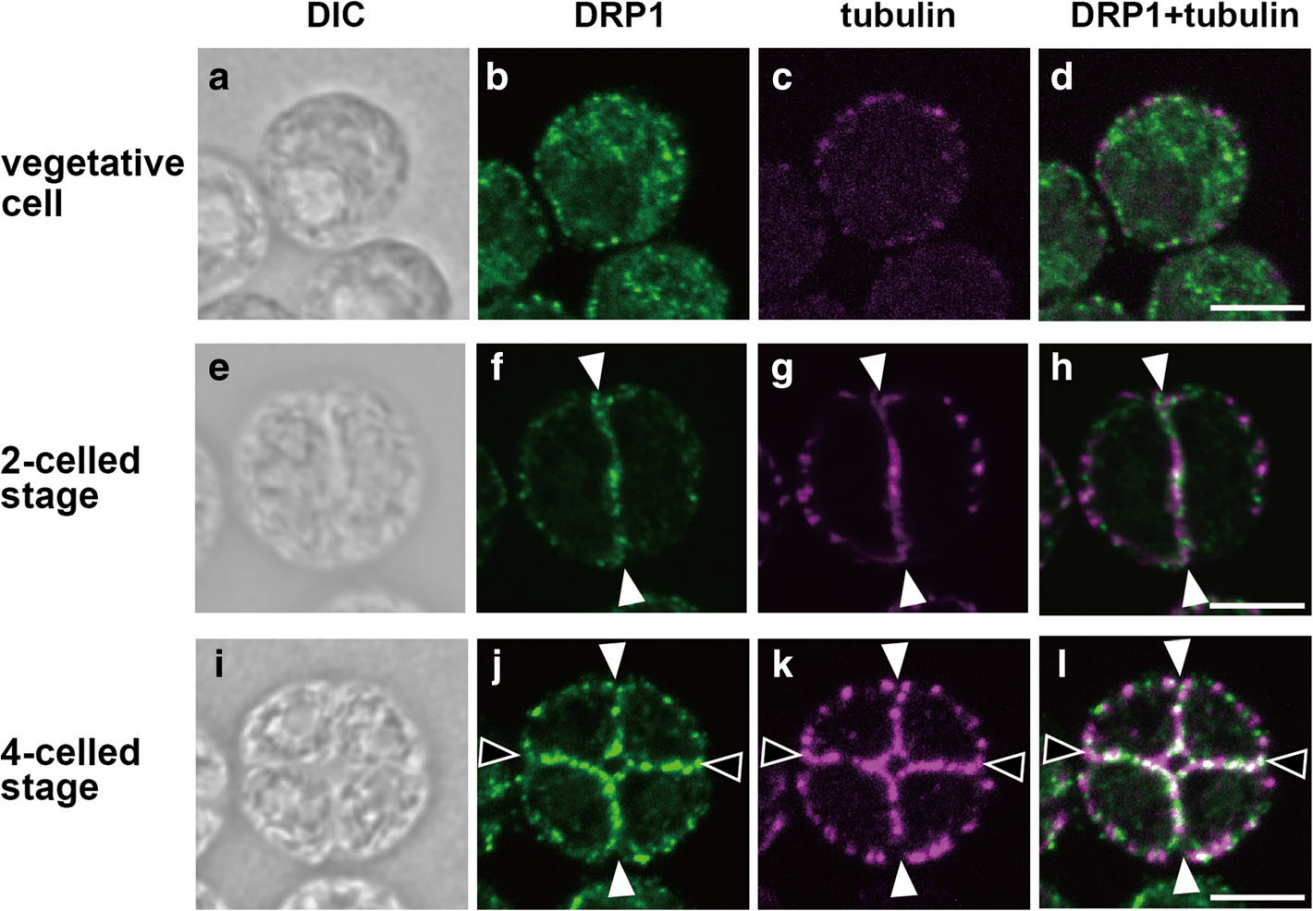
Immunofluorescence images of DRP1 localization in *Tetrabaena socialis.* Differential interference contrast (DIC) images (**a**, **e**, **i**), immunofluorescence images labeled with an anti-TsDRP1 (**b**, **f**, **j**) and an anti-tubulin alpha (**c**, **g**, **k**), and merged immunofluorescence images of DRP1 and tubulin (**d**, **h**, **l**) are shown. In vegetative cells, DRP1 was localized to cytoplasm (**b**-**d**). DRP1 was localized to a first division plane (white arrowheads in **f**-**h**) of two-celled embryo and both first (white arrowheads in **j**-**l**) and second (blank arrowheads in **j**-**l**) division planes of four-celled embryo. The first and second division planes in the four-celled stage were estimated by directions of the planes within the parental colony. Scale bars: 10 μm

**Fig. 5:**
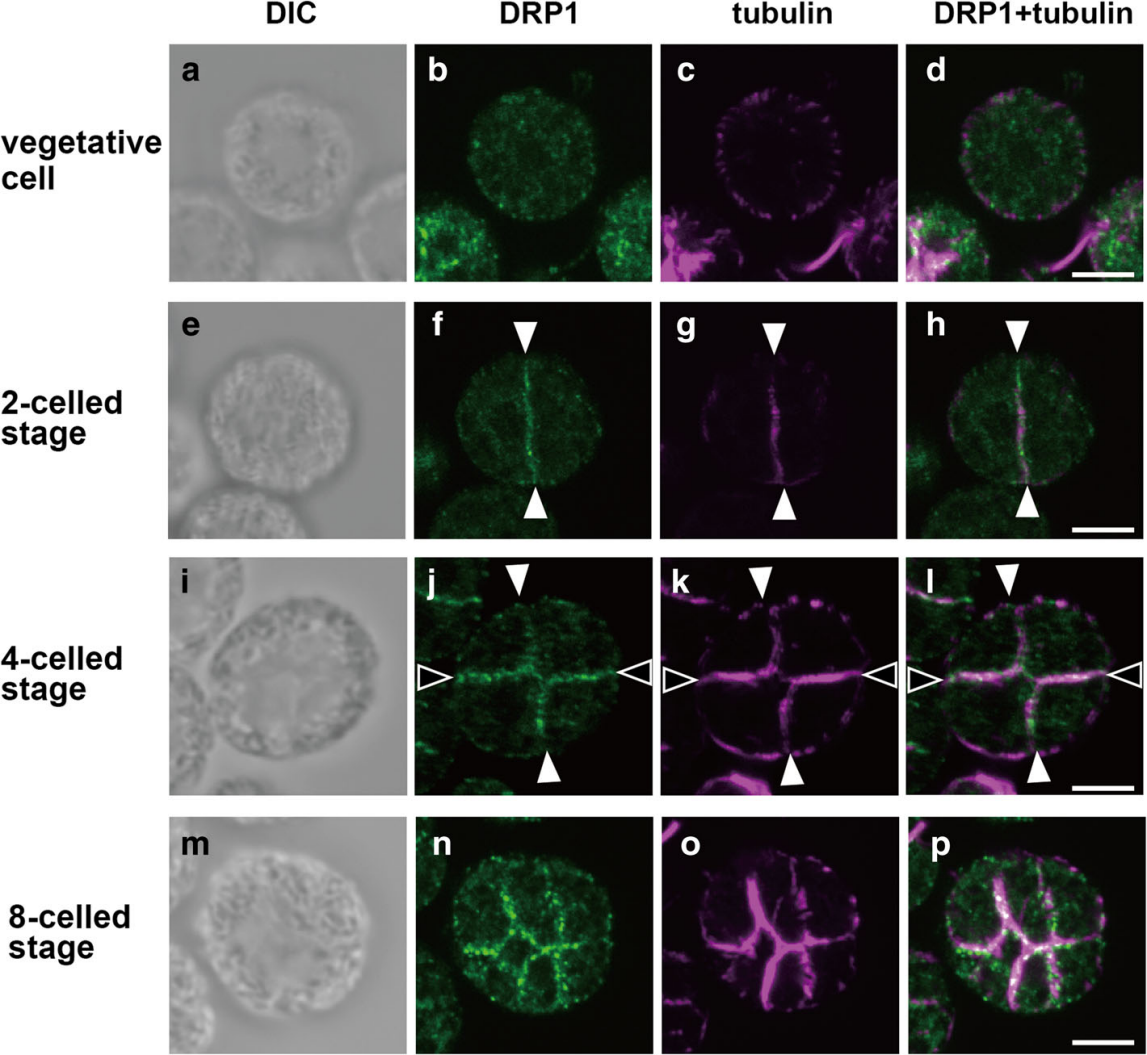
Immunofluorescence images of DRP1 localization in *Gonium pectorale*. Differential interference contrast (DIC) images (**a**, **e**, **i**, **m**), immunofluorescence images labeled with an anti-TsDRP1 (**b**, **f**, **j**, **n**) and an anti-tubulin alpha (**c**, **g**, **k**, **o**), and merged immunofluorescence images of DRP1 and tubulin (**d**, **h**, **l**, **p**) are shown. In vegetative cells, DRP1 was localized to cytoplasm (**a**-**d**). DRP1 was localized to a first division plane of two-celled embryo (white arrowheads in **f**-**h**), both first and second division planes of four-celled embryo (**j**-**l**), all first, second, third division planes of eight-celled embryo (**n**-**p**). The second division plane in four-celled stage was estimated by strong immunofluorescence of the anti-tubulin alpha (phycoplast). Scale bars: 10 μm

To verify the subcellular localization of DRP1, immunofluorescence microscopy was carried out using the anti-TsDRP1 and anti-tubulin alpha antibodies. Immunofluorescences of tubulin were used as the division plane marker, because microtubule structures (phycoplast) are observed in division planes of volvocine algae [39–41]. In vegetative cells of *C. reinhardtii*, *T. socialis* and *G. pectorale*, DRP1 signals were observed as many speckles in their cytoplasm (Figs. 3a-d, 4a-d, 5a-d). In two-celled stage, microtubules were observed in the cleavage furrows and DRP1 signals were localized in the vicinity of the division plane of *C. reinhardtii* (white arrowheads in Fig. 3e-h), *T. socialis* (white arrowheads in Fig. 4e-h) and *G. pectorale* (white arrowheads in Fig. 5e-h). While tubulin and DRP1 signals were not perfectly co-localized in the two-celled stage, these appeared to be partially overlapped (Figs. 3h, 4h, 5h). The DRP1 fluorescence in cytoplasm at this stage was less than that of vegetative cell (Figs. 3f, 4f, 5f).

DRP1 localizations of the four-celled stage were different between the unicellular and multicellular species examined here. In the four-celled stage of *C. reinhardtii*, DRP1 was mainly localized in second division planes (Fig. 3i-l). In contrast, DRP1 in four-celled embryos of *T. socialis* and *G. pectorale* were observed clearly in both first and second division planes (Figs. 4i-l, 5i-l). Eight-celled embryo of *G. pectorale* also exhibited clear DRP1 localization in the first, second and third division planes (Fig. 5m-p).

## Discussion

In *C. reinhardtii*, *T. socialis* and *G. pectorale*, DRP1 signals were mainly observed in their cytoplasm during the vegetative phase (Figs. 3b, 4b, 5b). However, the signals were mainly accumulated in the division plane during multiple fission of the three volvocine species (Figs. 3f, j, 4f, j, 5f, j, n). DRP1 expression was always detected and did not drastically change during the time courses examined in the three species (Additional file 2: Figure S3). Thus, volvocine DRP1 might change localization during the life cycle. The volvocine DRP1 localizations in the division plane were similar to those of some DRP1 proteins of land plants: DRP1A (ADL1) in *A. thaliana* [20] and tobacco BY-2 cells [42], and DRP1C in tobacco BY-2 cells [42]. Therefore, DRP1 proteins of volvocine algae may act as cytokinesis-related molecules.

In the second division of multiple fission (4-celled stage), DRP1 of *C. reinhardtii* was mainly localized in the second division plane (Fig. 3j), while DRP1 signals of *T. socialis* (Fig. 4j) and *G. pectorale* (Fig. 5j) were clearly observed in the both division planes, which were formed by the first and second divisions. Moreover, in the third division of *G. pectorale* (8-celled stage), DRP1 signals were observed in all division planes (Fig. 5n). Thus, DRP1 may have a role mainly in last division plane of unicellular *C. reinhardtii* (Fig. 3f, j), whereas DRP1 may equally work in all division planes of multicellular *T. socialis* and *G. pectorale* (Figs. 4f, j,5f, j, n). Each cell division during multiple fission of multicellular *T. socialis* [5], *G. pectorale* [12], and other multicellular volvocine algae [13, 43–45] proceeds through incomplete cytokinesis and the resulting daughter protoplasts are connected to one another by cytoplasmic bridges, whereas daughter protoplasts of *C. reinhardtii* are completely separated from one another by means of complete cytokinesis [9, 10]*.* The division planes of multicellular volvocine species are therefore different from those of unicellular *C. reinhardtii* at subcellular and molecular levels.

Dynamins and DRPs are involved in various membrane remodeling events such as budding and trafficking of vesicles, fission or fusion of organelles, and cytokinesis [22, 46]. Particularly in cytokinesis, dynamins are associated with newly formed membranes of metazoans such as *Caenorhabditis elegans* [47] and zebrafish [48]. In *Dictyostelium discoideum*, the relationships between cytokinesis and dynamin A [49], dynamin-like protein (Dlp) A, DlpB and DlpC [22] were reported. In *A. thaliana*, DRP1A [20], DRP1E [20], DRP2B [21], and DRP5A [22] are localized in the division plane, and function in cytokinesis. Those dynamins and DRPs play important roles for cytokinesis such as membrane fission and vesicle formation for cytokinesis. Therefore, volvocine DRP1 proteins might be related to membrane remodeling during the multiple fission. Hence, the few DRP1 signals in the first division plane of *C. reinhardtii* 4-celled stage (Fig. 3j) may indicate that membrane fission/formation mediated by DRP1 has been finished in this plane after the first division (white arrowheads in Fig. 3j), but has been occurring at the second division plane (black arrowheads in Fig. 3j). The presence of DRP1 signals in all division planes of *T. socialis* (Fig. 4j) and *G. pectorale* (Fig. 5j, n) may be due to the continuous membrane remodeling during the incomplete cytokinesis in these two multicellular species.

## Conclusion

**Fig. 6:**
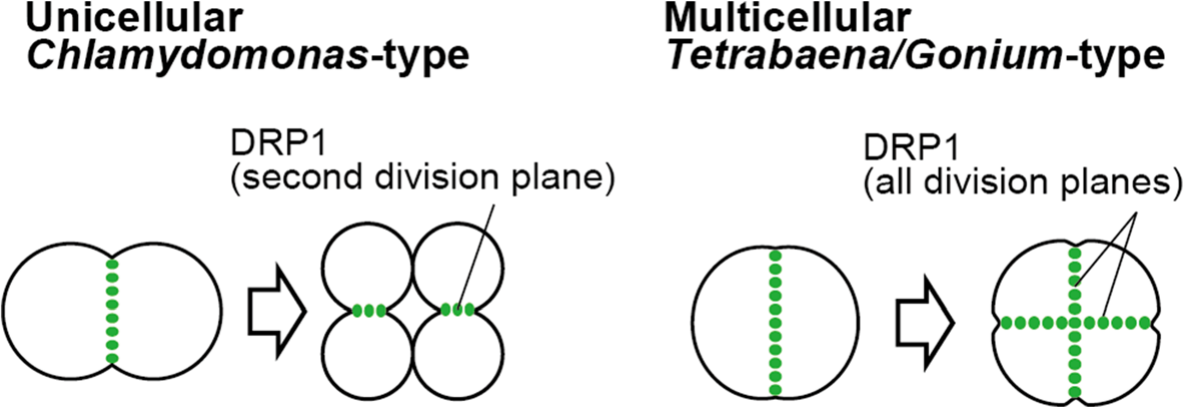
Schematic diagram of DRP1 localization patterns in volvocine algae. In unicellular *Chlamydomonas reinhardtii*, DRP1 is mainly localized to second division planes of four-celled stage (unicellular *Chlamydomonas*-type). In multicellular *Tetrabaena socialis* and *Gonium pectorale*, DRP1 is localized to all division planes of four-celled embryo (multicellular *Tetrabaena*/*Gonium*-type)

This study demonstrated that localization patterns of DRP1 are different between unicellular *C. reinhardtii* and two multicellular species *T. socialis* and *G. pectorale*. Given that DRP1 may function in volvocine cytokinesis, the different DRP1 localization patterns between *C. reinhardtii* and two multicellular species *T. socialis* and *G. pectorale* (Fig. 6) indicate differences in the molecular mechanisms of cytokinesis during multiple fission between unicellular forms and multicellular forms. *T. socialis* and *G. pectorale* are considered to represent ancestral (plesiomorphic) multicellular morphology based on the cladistic analysis of morphological data and molecular phylogenetic analyses (Fig. 1) [3–7]. These data indicate therefore, that the localization patterns of DRP1 during multiple fission might have been modified from unicellular *Chlamydomonas*-type to multicellular *Tetrabaen*a/*Gonium*-type (Fig. 6) in the common ancestor of multicellular volvocine algae (Fig. 1). This modification was essential for the initial stages of colonial living in this lineage.

## Additional files

Additional file 1 **Table S1.** List of primers used for amplification and sequencing of *TsDRP1*. **Table S2.** List of DRP1 and DRP2 proteins used in this study. (PDF 84 kb)

Additional file 2 **Figure S1.** Alignment of DRP1A from * Arabidopsis thaliana* (At) and DRP1 from *Chlamydomonas reinhardtii* (Cr), *Tetrabaena socialis* (Ts), *Gonium pectorale* (Gp) and *Volvox carteri* (Vc). Black and gray background indicates identical or similar amino acid, respectively. GTPase domain, dynamin middle domain, and GTPase effector domain are indicated by pink, green, and yellow background color, respectively. The region corresponding to the antigen for an anti-TsDRP1 antibody is showed under the alignment (gray bar). **Figure S2.** Specificity of the affinity-purified anti-TsDRP1 antibody. The specificity of the anti-TsDRP1 antibody was validated in three volvocine algae by western blotting. A single band was detected in each lane (~75 kDa) with the antibody that was incubated with acetone powder of *E. coli* with the empty vector (left) while no signal was detected with the antibody that was incubated with acetone powder of *E. coli* expressing TsDRP1 (middle). For details of the methods, see Information S1 (Additional file 2). **Figure S3.** Western blot analyses of DRP1 proteins of *Chlamydomonas reinhardtii* (CrDRP1), *Tetrabaena socialis* (TsDRP1), and *Gonium pectorale* (GpDRP1) using anti-TsDRP1 antibody. Time-course of synchronous culture and western blot (WB) of *C. reinhardtii*, *T. socialis*, and *G. pectorale* are shown in **a**, **b**, and **c**, respectively. Time-course samples were obtained from five points (arrows in each line graph): the greatest number of dividing cells (0), three (−3) and six (−6) hours before 0 point, and three (+3) and six (+6) hours after 0 point. Coomassie brilliant blue (CBB) staining of a duplicate gel shows the equal protein loading in each lane. **Information S1. **Methods for specificity of the affinity-purified anti-TsDRP1 antibody (Additional file 2: Figure S2). (PDF 1785 kb)

## Abbreviations

DIC: Differential interference contrast
Dlp: Dynamin-like protein
DRP: Dynamin-related protein
GED: GTPase effector domain
GTP: Guanosine triphosphate
GTPase: Guanosine triphosphatease
PBS: Phosphate-buffered saline
TPBS: 0.1% Tween 20 in PBS
