# Supplementary material for: Evolution of cytokinesis-related protein localization during the emergence of multicellularity in volvocine green algae
Source: BMC Evol Biol. 2017 Dec 6;17:243. doi: 10.1186/s12862-017-1091-z (PMC5717801; doi:10.1186/s12862-017-1091-z)
Supplement: Supplementary file 1 — Table S1. List of primers used for amplification and sequencing of TsDRP1. Table S2. List of DRP1 and DRP2 proteins used in this study. (PDF 84 kb) [file 12862_2017_1091_MOESM1_ESM.pdf]

**Additional file 1:**

**Tables S1, S2**

**Table S1.** List of primers used for amplification and sequencing of *TsDRP1*.

| Primer Designation | Sequences (5'-3') |
|---|---|
| CrDRP1_R3 | GTCCATAATGTCCACCTTGGTC |
| CrDRP1_R9 | GATCTCCTCGCTCTCGTTGAT |
| TsDRP1_F1 | GTGATCGGACTCGTCAACAA |
| TsDRP1_F3 | AAGTCGTCGGTGCTGGAG |
| TsDRP1_F4 | GCAGTACATCAAGAGCGACAAC |
| TsDRP1_F12 | CGAGTTCTTCCAGAGCAAGC |
| TsDRP1_R2 | GTGCGCGAACTCCCCATAGT |
| TsDRP1_R14 | ACACGTCCAGGATCTTCTCG |
| TsDRP1_3'UTR_F1 | TCAACGGCGAGAACCTGTTCGAGAAGC |
| TsDRP1_3'UTR_F2 | CTGCTGACGGACCTGCAGGAGGAGAC |
| TsDRP1_5'UTR_R1 | GGAGCTCTGTCCACCGACAACGACGAT |
| TsDRP1_5'UTR_R2 | ATGGTTGGGAGCTTGCTCCACAGGATG |
| TsDRP1_antigen_F1[a] | CACCCTGCAGCAAATTTGCACGTCGCTC |
| TsDRP1_antigen_R2[a] | TTAGAGGCGCCGCAGCTGCGGCTCCTC |

[a]Primers for antigen.

**Table S2.** List of DRP1 and DRP2 proteins used in this study.

| Species | Protein Name | Locus Name/Accession Number |
|---|---|---|
| *Chlamydomonas reinhardtii* | DRP1 | Cre05.g245950.t1.1[b] |
| *Tetrabaena socialis* | DRP1 | LC279615 [a] |
| *Gonium pectorale* | DRP1 | KXZ46173[c] |
| *Volvox carteri* | DRP1 | Vocar.0026s0065[b] |
| *Coccomyxa subellipsoidea* | Dynamin family | estExt_fgenesh1_pm.C_190089[b] |
| *Micromonas pusilla* | DYNAMIN | e_gw1.14.510.1[b] |
| *Micromonas* sp. | Dynamin family | EuGene.1500010100[b] |
| *Arabidopsis thaliana* | DRP1A[a] | AT5G42080[b] |
| | DRP1B[a] | AT3G61760[b] |
| | DRP1C[a] | AT1G14830[b] |
| | DRP1D[a] | AT2G44590[b] |
| | DRP1E[a] | AT3G60190[b] |
| | DRP2A[a] | AT1G10290[b] |
| | DRP2B[a] | AT1G59610[b] |
| *Oryza sativa* | Os02 g50550[a] | LOC_Os02g50550[b] |
| | Os03 g50520[a] | LOC_Os03g50520[b] |
| | Os05 g48240[a] | LOC_Os05g48240[b] |
| | Os06 g13820[a] | LOC_Os06g13820[b] |
| | Os08 g32920[a] | LOC_Os08g32920[b] |
| | Os09 g39960[a] | LOC_Os09g39960[b] |
| | Os10 g41820[a] | LOC_Os10g41820[b] |
| *Amborella trichopoda* | DRP1A | evm_27.TU.AmTr_v1.0_scaffold00021.256[b] |
| | DRP1C | evm_27.TU.AmTr_v1.0_scaffold00176.26[b] |
| | DYNAMIN | evm_27.TU.AmTr_v1.0_scaffold00002.113[b] |
| | DRP2A | evm_27.TU.AmTr_v1.0_scaffold00080.49[b] |
| *Selaginella moellendorffii* | DRP1C-1 | 183216[b] |
| | DRP1C-2 | 266589[b] |

| | | |
|---|---|---|
| | DRP1C-3 | 90013[b] |
| | DRP2A-1 | 171046[b] |
| | DRP2A-2 | 77824[b] |
| *Physcomitrella patens* | DRP1C-1 | Pp3c18_20531[b] |
| | DRP1C-2 | Pp3c19_3590[b] |
| | DRP1C-3 | Pp3c19_4870[b] |
| | DRP1C-4 | Pp3c22_1100[b] |
| | DRP1C-5 | Pp3c22_2500[b] |
| | DRP2A-1 | Pp3c18_20600[b] |
| | DRP2A-2 | Pp3c19_3640[b] |
| | DRP2A-3 | Pp3c1_8440[b] |
| | DRP2A-4 | Pp3c2_32060[b] |
| *Klebsormidium flaccidum* | DLP-1[c] | GAQ83701[b] |
| | DRP[c] | GAQ87226[b] |

[a]Determined in this study.

[b]Obtained from Phytozome (https://phytozome.jgi.doe.gov/pz/portal.html).

[c]Obtained from National Center for Biotechnology Information (NCBI) database (https://www.ncbi.nlm.nih.gov).
