# Supplementary material for: Evolution of cytokinesis-related protein localization during the emergence of multicellularity in volvocine green algae
Source: BMC Evol Biol. 2017 Dec 6;17:243. doi: 10.1186/s12862-017-1091-z (PMC5717801; doi:10.1186/s12862-017-1091-z)
Supplement: Supplementary file 2 — Figure S1. Alignment of DRP1A from Arabidopsis thaliana (At) and DRP1 from Chlamydomonas reinhardtii (Cr), Tetrabaena socialis (Ts), Gonium pectorale (Gp) and Volvox carteri (Vc). Black and gray background indicates identical or similar amino acid, respectively. GTPase domain, dynamin middle domain, and GTPase effector domain are indicated by pink, green, and yellow background color, respectively. The region corresponding to the antigen for an anti-TsDRP1 antibody is showed under the alignment (gray bar). Figure S2. Specificity of the affinity-purified anti-TsDRP1 antibody. The specificity of the anti-TsDRP1 antibody was validated in three volvocine algae by western blotting. A single band was detected in each lane (~75 kDa) with the antibody that was incubated with acetone powder of E. coli with the empty vector (left) while no signal was detected with the antibody that was incubated with acetone powder of E. coli expressing TsDRP1 (middle). For details of the methods, see Information S1 (Additional file 2). Figure S3. Western blot analyses of DRP1 proteins of Chlamydomonas reinhardtii (CrDRP1), Tetrabaena socialis (TsDRP1), and Gonium pectorale (GpDRP1) using anti-TsDRP1 antibody. Time-course of synchronous culture and western blot (WB) of C. reinhardtii, T. socialis, and G. pectorale are shown in a, b, and c, respectively. Time-course samples were obtained from five points (arrows in each line graph): the greatest number of dividing cells (0), three (−3) and six (−6) hours before 0 point, and three (+3) and six (+6) hours after 0 point. Coomassie brilliant blue (CBB) staining of a duplicate gel shows the equal protein loading in each lane. Information S1. Methods for specificity of the affinity-purified anti-TsDRP1 antibody (Additional file 2: Figure S2). (PDF 1785 kb) [file 12862_2017_1091_MOESM2_ESM.pdf]

Additional file 2:

Figures S1-S3

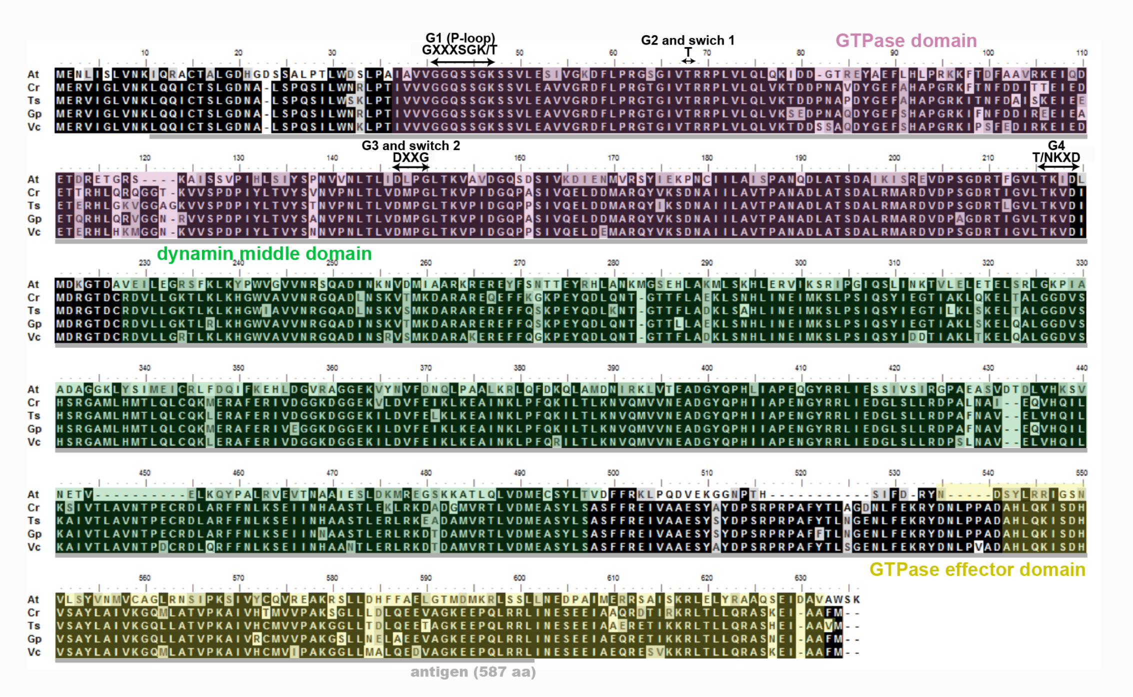

Figure S1. Alignment of DRP1A from *Arabidopsis thaliana* and DRP1 from four volvocine algae. Deduced amino acid sequences of DRP1A from *A. thaliana* (At) and DRP1 from *Chlamydomonas reinhardtii* (Cr), *Tetrabaena socialis* (Ts), *Gonium pectorale* (Gp) and *Volvox carteri* (Vc) were aligned. Black and gray background indicates identical or similar amino acid, respectively. GTPase domain, dynamin middle domain, and GTPase effector domain are indicated by pink, green, and yellow background color, respectively. The region corresponding to the antigen for an anti-TsDRP1 antibody is showed under the alignment (gray bar).

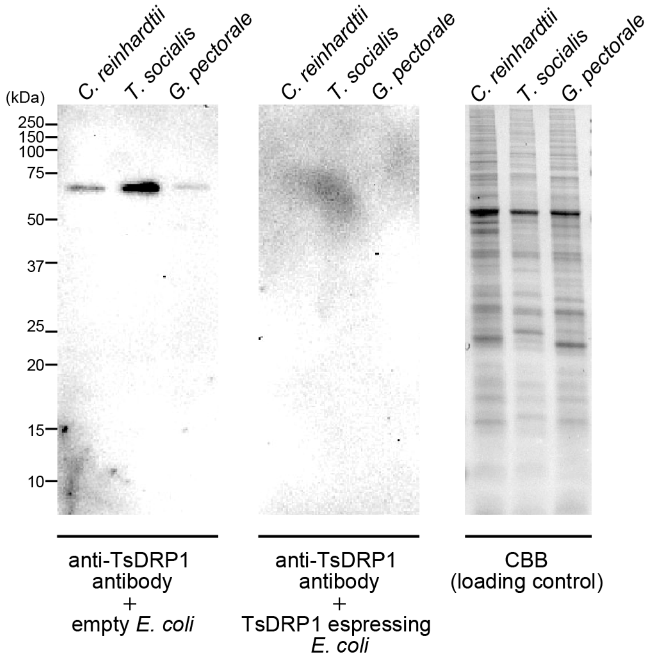

Figure S2. Specificity of the affinity-purified anti-TsDRP1 antibody. The specificity of the anti-TsDRP1 antibody was validated in three volvocine algae, *Chlamydomonas reinhardtii*, *Tetrabaena socialis*, and *Gonium pectoral* by western blotting. The antibody (1:2,000) was diluted in 3% skim milk dissolved in TTBS. 1 mL of the antibody solution was incubated with 300 µL of acetone powder of *E. coli* with empty pET100 vector or *E. coli* expressing TsDRP1 by pET100 vector overnight at 20˚C. The acetone powder was prepared as follows. *E. coli* BL21 with the empty pET100 or pET100- TsDRP1 was cultured in 500 mL of LB medium at 37˚C and 1 mM IPTG was added when OD750 reached ~0.5. The culture was further incubated at 37˚C for 2 h. The cells were harvested by centrifugation and resuspended in 10 mL of 20 mM Tris-HCl, pH 8.0 supplemented with 0.1 µg/mL lysozyme at 37˚C for 2 h. After the lysozyme treatment, 30 mL of acetone was added and the mixture was centrifuged at 10,000 *g* for 20 min. The pellet was air dried and used as acetone powder. A single band

was detected in each lane (~75 kDa) with the antibody that was incubated with acetone powder of *E. coli* with the empty vector (left) while no signal was detected with the antibody that was incubated with acetone powder of *E. coli* expressing TsDRP1 (middle). Coomassie Brilliant Blue (CBB) staining of a duplicate gel is also shown as a loading control (right).

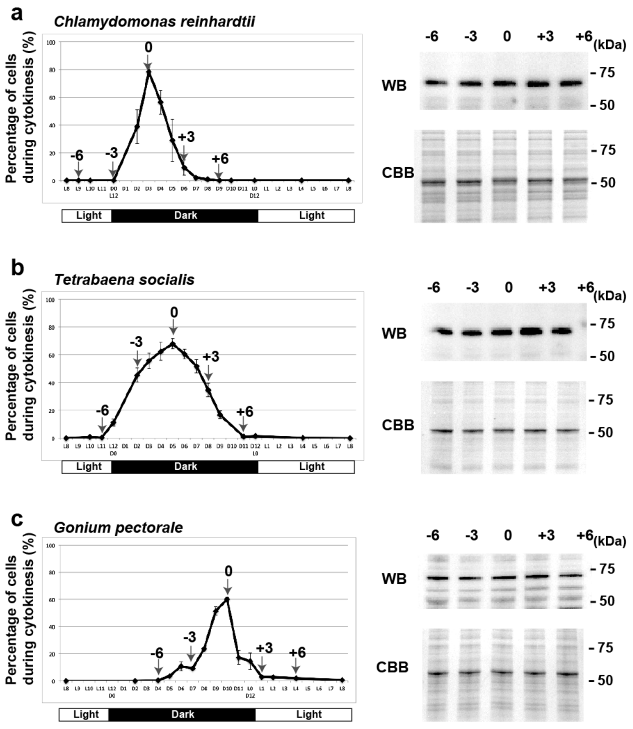

Figure S3. Western blot analyses of volvocine DRP1. DRP1 of *Chlamydomonas reinhardtii* (CrDRP1), *Tetrabaena socialis* (TsDRP1), and *Gonium pectorale* (GpDRP1) was detected by the anti-TsDRP1 antibody. Time-course of synchronous culture and western blot (WB) of *C. reinhardtii*, *T. socialis*, and *G. pectorale* are shown in **a**, **b**, and **c**, respectively. Time-course samples were obtained from five points (arrows in each line graph): the greatest number of dividing cells (0), three (-3) and six (-6) hours before 0 point, and three (+3) and six (+6) hours after 0 point. Coomassie brilliant blue (CBB) staining of a duplicate gel shows the equal protein loading in each lane.
